# Electronic bypass of spinal lesions: activation of lower motor neurons directly driven by cortical neural signals

**DOI:** 10.1186/1743-0003-11-107

**Published:** 2014-07-03

**Authors:** Yan Li, Monzurul Alam, Shanshan Guo, KH Ting, Jufang He

**Affiliations:** 1 Department of Rehabilitation Sciences, The Hong Kong Polytechnic University, Hung Hom, Kowloon, Hong Kong; 2 Department of Biomedical Sciences, City University of Hong Kong, Tat Chee Avenue, Kowloon, Hong Kong; 3 Department of Neurosurgery, University of California, Los Angeles, CA, USA

**Keywords:** Spinal cord injury, Multielectrode array, Extracellular recording, Neural spikes, Functional electrical stimulation, Intraspinal microstimulation, Intracortical microstimulation, Locomotion, Neuromotor prostheses

## Abstract

**Background:**

Lower motor neurons in the spinal cord lose supraspinal inputs after complete spinal cord injury, leading to a loss of volitional control below the injury site. Extensive locomotor training with spinal cord stimulation can restore locomotion function after spinal cord injury in humans and animals. However, this locomotion is non-voluntary, meaning that subjects cannot control stimulation via their natural *“intent”*. A recent study demonstrated an advanced system that triggers a stimulator using forelimb stepping electromyographic patterns to restore quadrupedal walking in rats with spinal cord transection. However, this indirect source of *“intent”* may mean that other non-stepping forelimb activities may false-trigger the spinal stimulator and thus produce unwanted hindlimb movements.

**Methods:**

We hypothesized that there are distinguishable neural activities in the primary motor cortex during treadmill walking, even after low-thoracic spinal transection in adult guinea pigs. We developed an electronic spinal bridge, called *“Motolink”*, which detects these neural patterns and triggers a “spinal” stimulator for hindlimb movement. This hardware can be head-mounted or carried in a backpack. Neural data were processed in real-time and transmitted to a computer for analysis by an embedded processor. Off-line neural spike analysis was conducted to calculate and preset the spike threshold for “*Motolink*” hardware.

**Results:**

We identified correlated activities of primary motor cortex neurons during treadmill walking of guinea pigs with spinal cord transection. These neural activities were used to predict the kinematic states of the animals. The appropriate selection of spike threshold value enabled the *“Motolink”* system to detect the neural *“intent”* of walking, which triggered electrical stimulation of the spinal cord and induced stepping-like hindlimb movements.

**Conclusion:**

We present a direct cortical *“intent”*-driven electronic spinal bridge to restore hindlimb locomotion after complete spinal cord injury.

## Background

Lower motor neurons (LMNs) and interneurons lose supraspinal controls after complete spinal cord injury (SCI). Although the restoration of volitional control of paralyzed limbs after complete SCI remains challenging, intracortical recording and functional electrical stimulation (FES) techniques have been successful in many studies [1-8]. Research in regenerative medicine has demonstrated the reconnection of corticospinal neurons in adult mice, which provides great hope for functional recovery after SCI [8-11]. Stem cell research also suggests that paralyzed patients could regain the ability to move after trauma [12,13]. However, such research has been limited to animal experiments or single human trials, and no treatment approaches are currently ready for clinical implementation in human patients [14,15]. Although FES has been used to activate paralyzed muscles to restore movements such as hand grasp, standing up, and taking a few steps, this stimulation is externally driven and does not arise from internal *“intent”* signals. Furthermore, FES may cause muscle fatigue after chronic use [16,17]. However, the activation of motor neurons in the spinal cord through intraspinal microstimulation (ISMS) can induce standing and walking in cats after SCI without producing major fatigue [18-22]. Also, tonic electrical stimulation of the epidural spinal cord has enabled a human patient to stand and take a couple of steps [8].

As an alternative approach to SCI treatment, neuromotor prostheses use cortical motor activities to control external devices to replace lost function [23-29]. A multichannel microelectrode array implanted in the cortex permits recording of neuronal signals—particularly motor command signals—from a patient’s brain, which are sent to a prosthetic device that can move a computer cursor [30] or a robotic arm to perform elementary actions such as self-feeding [31]. Excitingly, recent studies in monkeys show that cortical signals can be transformed to trigger muscle stimulation, leading to the restoration of goal-directed movements in transiently paralyzed arms [32,33]. These previous studies have mainly focused on the restoration of upper limb functions, with few studies attempting to restore locomotion [34-38].

In particular, Garasimenko et al. [39] demonstrated quadrupedal walking in cats after epidural electrical stimulation of the spinal cord with no input from the brain after complete spinal transection. This groundbreaking work was followed by several successful experiments in which locomotor function was similarly enhanced in both rats [40-43] and humans [44]. However, all of these studies required an external device to operate the stimulator. By contrast, a very recent study demonstrated an advanced system that triggers the stimulator using forelimb electromyographic (EMG) patterns to restore quadrupedal walking in rats with spinal cord transection [45]. Though epidural stimulation represented robust muscles activation in previous investigations [8,39,41,43,45], intraspinal stimulation has shown better efficacy and selectivity of muscles activation than epidural stimulation [46,47]. Other researchers propose that stepping movements after SCI could be controlled with one or two independent cortical signals using ISMS with an assistive computer system [48].

In the present study, we tested the feasibility of triggering stimulation of LMNs in the spinal cord from direct cortical neural recordings in guinea pigs after complete mid-thoracic spinal cord transection. As such we also evaluated the feasibility of utilizing a new animal model, as the restoration of walking has already been demonstrated in cats and rats. Finally, we developed an electronic spinal bridge, called *“Motolink”*, which bypasses the spinal cord lesion. This *“Motolink”* hardware is comprised of low-noise, high-gain recording amplifiers and a programmable neural processor in a surface-mounted stimulator circuit board, which can be mounted on the head or carried on a backpack by small animal subjects.

## Methods

**Figure 1:**
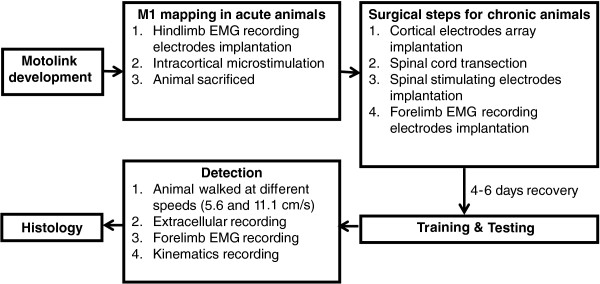
Flow chart of the series of experiments and procedures.

Experiments were conducted in compliance with the *Principles of Laboratory Animal Care* (National Institutes of Health, No. 86–23, revised 1985), and all experimental procedures were approved by the Animal Subjects Ethics Sub-Committee at The Hong Kong Polytechnic University. The flow chart of the series of experiments provides detailed explanation (Figure 1).

### Hardware development

**Figure 2:**
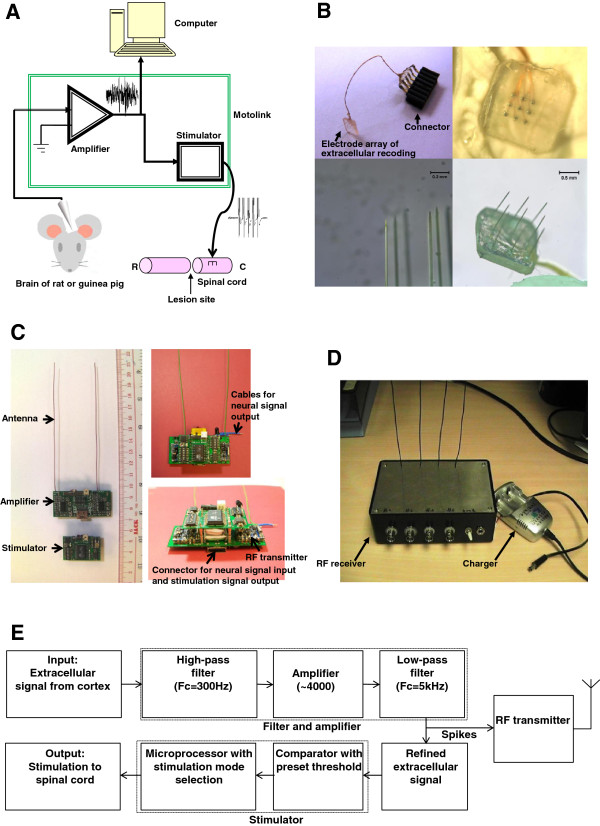
**Schematic diagram and overview of electronic *“Motolink” *spinal bridge. A**. Conceptual diagram. **B**. Electrodes array for extracellular recording. **C**. Wireless hardware. **D**. Remote signal receiver. **E**. Hardware block diagram. Neural signals undergo three stages of amplification and filtering and are then compared to a preset threshold for generation of spinal cord stimulation.

An electronic spinal bridge, called *“Motolink”*, was developed in the Laboratory of Applied Neuroscience at The Hong Kong Polytechnic University. *“Motolink”* is a miniature prosthesis that amplifies neuronal spikes, compares them to pre-calculated and preset thresholds, and converts the signals into stimulation pulses using a microprocessor-based chip (89C2051, ATMEL, San Jose, CA, USA; Figure 2A). Intracortical microelectrode arrays recorded extracellular neural spikes (Figure 2B), which were amplified and decoded to generate stimulation patterns that were applied below the lesion of the spinal cord. Amplifier and stimulator circuits were combined (Figure 2C), although they received separate power supplies to avoid interference. Another amplifier was attached to the stimulator and recorded EMG signals from the left forelimb. Dipswitches placed on the circuit for multi-choice selection of stimulation pattern and enabled linkage between the recording and stimulating channels. Amplification ranges from 1 to 10,000 in theory but was set at ~4,000 in practice. This amplification was sufficient for recording action potentials from the cortex and EMG signals from skeletal muscles. Neural signals were applied and detected from the analog form. Five ms after detection of a supra-threshold signal, five monophasic stimulation pulses (91 Hz) with variable amplitudes (3–7 V) were generated.The system was comprised of different cascading modules of amplifiers and filters with an effective frequency bandwidth between 300 Hz and 5 KHz, followed by a smoother and microprocessor-based neural stimulation circuit (Figure 2E). Raw spikes were transmitted by a radio frequency transmitter module to a host computer for real-time analysis. Neural and EMG signals were sent to the computer with a sampling rate of 10 kHz and received by a Radio Frequency receiver (Figure 2D).

### Primary motor cortex mapping

Before surgical implantation, motor cortex mapping was performed to determine the electrode array implantation site in acute animals. Four adult Hartley albino guinea pigs were used to map the primary motor cortex (M1) in the right hemisphere using intracortical microstimulation (ICMS) [49,50]. Animals were mounted in a stereotaxic device, and body temperature was controlled by a homoeothermic system. To obtain a fine map of the hindlimb region of the M1, we monitored EMG signals from the gluteus superficialis, bicep femoris, semitendinosus, and tibialis anterior muscles. Under anesthesia (sodium pentobarbital, 40 mg/kg i.p. initially and 10 mg/kg/h afterward; Ceva Sante Animale Co., France), four wires with exposed tips (1–2 mm) were inserted into muscle bellies and served as EMG recording electrodes [51]. The wires were sutured at their entrance into muscle bellies and looped around the entrance site to relieve stress. Craniotomy was performed to expose the right M1. After carefully removing the dura matter, a low impedance tungsten electrode (10–100 kΩ, FHC Inc., Bowdoin, ME) was slowly advanced into the cortex using a micromanipulator. Stimulation pulses were delivered with respect to a reference electrode placed on the scalp, and evoked motor responses were carefully observed. We slowly varied current intensity to find a threshold that could evoke motor responses (13-pulse train, 0–250 μA, 333 Hz, generated by TDT system, Alachua, FL). When the contralateral forelimb/trunk/hindlimb responded to the stimulation, that stimulation site was considered positive. After that, the electrode was inserted into another site adjacent and repeated the stimulation procedure until the whole map was achieved. At the end of the experiment, guinea pigs were sacrificed by overdose of pentobarbital sodium (60 mg/kg, i.p.).

### Animal preparation and surgery

Based on the results of M1 mapping performed in acute animals, surgery under aseptic conditions was performed on chronic adult guinea pigs (Caviaporcellus, 400–800 g, both sexes, SPF). Animals were pretreated with atropine (40–200 pg/kg, s.c.; Sigma, USA) to reduce respiratory secretions. Anesthesia was initiated with pentobarbital sodium (40 mg/kg, i.p.) and maintained by supplemental doses (10 mg/kg/hr, i.p.). Animals were mounted in a stereotaxic device, and body temperature was controlled by a homoeothermic system. Craniotomy was performed to access the right M1. After careful removal of the dura matter, the hindlimb region of the right M1 was identified through visual observation of left hindlimb movement in response to intracortical electrical stimulation. An electrode array was implanted into the hindlimb region of the right M1 with minimal lesion using a pneumatically actuated microelectrode array inserter (Blackrock Microsystems, UT, USA). The center of the recording electrode array was located at −2.3 mm AP relative to bregma and 2.0 mm ML relative to the midline. The array was comprised of 2 × 3 or 3 × 3 Teflon-coated tungsten electrodes (50 μm diameter, 200–500 kΩ impedance) with 0.5-mm spaces between electrodes (SM Tang’s group, Institute of Biophysics, Chinese Academy of Sciences; Figure 2B). The electrode tips were exposed by 10–30 μm. A stainless steel reference electrode (Teflon-coated stainless steel, 1 mm exposed tip, A-M Systems, USA) was placed on the surface of a non-M1 area of the cortex in the right hemisphere. The opening of the skull was covered with silicone (World Precision Instruments, USA). Five screws were placed in the skull, with a ground wire wrapped around one of the screws. The electrode array and ground wire were connected to a socket. The electrode array, screws, wires, lower portion of the socket, and exposed skull were all covered by dental cement.

The animal’s body was lifted up and fixed by two clips on the spinous process at lumbar and sacral levels. Laminectomy was performed to expose the T12 and L2-L3 spinal cord. T12 was carefully transected with microscissors. Care was taken not to damage the spinal arteries. A reference stimulating electrode (Teflon-coated stainless steel wire, 225 μm diameter, 25 kΩ impedance, 1 mm exposed tip, A-M Systems, USA) was placed on the epidural surface near L5 or in the back muscles close to the lumbar spine. After carefully removing the dura matter, five stimulating electrodes (Teflon-coated stainless steel wire, 75 μm diameter, 50–100 kΩ impedance, cross-section exposed tips, A-M Systems, USA) were manually implanted vertically into the left ventral horn at level L2-L3 with electrical stimulation to target the LMNs 1.0–1.5 mm below the surface of the spinal cord. After confirming their location, microwires were embedded in silicone elastomer for fixation. All stimulating wires were led subcutaneously to the head socket.

Because we assumed that forelimb stepping was initiated by the cortical *“intent”* signals in the forelimb region of M1 during treadmill walking, we recorded forelimb EMG. Two EMG recording electrodes (Teflon-coated stainless steel, 50 μm diameter, 30–100 kΩ impedance, A-M Systems, USA) were inserted and sutured into the bellies of left forelimb muscle triceps brachii. An EMG reference electrode (Teflon-coated stainless steel, 225 μm diameter, 25 kΩ impendance, 1 mm exposed tip, A-M Systems, USA) was sutured on the tendon of triceps brachii near the elbow joint. All EMG electrodes were led subcutaneously to the head socket. The spinal opening and forelimb incision site were sutured.

After electrode implantation, animals were given buprenorphine (0.1 mg/kg i.s., twice a day for 3 days) and penicillin (50,000 units/kg i.m., once a day in case of infection). Recovery was carefully monitored for 4–6 days before starting treadmill experiments.

### Experimental procedure

Guinea pigs were placed on a treadmill with a custom-made harness. The hind part of the body was lifted with a bodyweight support system, thus allowing only forelimb steps. *“Motolink”* hardware was plugged into the head socket during experiments. Firstly, we turned on the amplifier circuits but turned off the stimulator circuit to collect signals without stimulation. Neural and forelimb EMG signals were recorded while animals walked at different speeds (5.6 and 11.1 cm/s). Signals were fed into a computer through a data acquisition system (AxoDigiData 1440, Molecular Devices Co., Chicago, IL). Neural activity in the hindlimb area of the M1 was filtered at 300 Hz to 3 kHz, and forelimb EMG signals were filtered at 30 Hz to 500 Hz. The onset of neural and EMG signals were determined by previously described methods [52]. The triggering threshold was calculated and preset to achieve a reasonable correlation between left forelimb and hindlimb movements. Then we turned on the stimulator circuit to enabled linkage between the recording and stimulating channels.

Kinematics of treadmill walking were recorded by a Vicon system (Vicon Version 370, three camera system, operating at 60 Hz, LA, CA) and a video camera (720 × 576 resolution, GZ-MG27AH, JVC, Japan). Retro-reflective markers (10 mm diameter) were attached to the skin of the left forelimb and left hindlimb for offline analysis.

### Histology

After experiments, animals were deeply anesthetized with pentobarbital sodium (60 mg/kg, i.p.; Sigma) and perfused transcardially with 400 ml 0.9% NaCl followed by ice-cold 4% paraformaldehyde in 0.1 M phosphate buffer (pH 7.4). The brain and spinal cord were removed and post-fixed for 4 h in the same fixative. The brain and spinal cord were cut into coronal sections (40 μm), and the spinal lesion was cut into horizontal sections. Nissl staining of sections was performed.

### Data analysis

Data were analyzed offline using custom scripts written in MATLAB (MathWorks, Nitick, MA). Extracellular data were processed by Axon Clampfit 10.0 (Molecular Devices Co., Chicago, IL). Neural spikes were detected and sorted using a MATLAB-based open source electrophysiological data processing toolbox [53]. Raster plots and spike time histograms were generated for each detected unit. Neural signals were compared with EMG signals and Vicon data using custom-written MATLAB scripts. Student's paired *t*-test was performed in each subject to compare the time lags between hindlimb and forelimb movements at different speeds. Data are shown as mean and standard error (SE).

## Results

### M1 mapping

**Figure 3:**
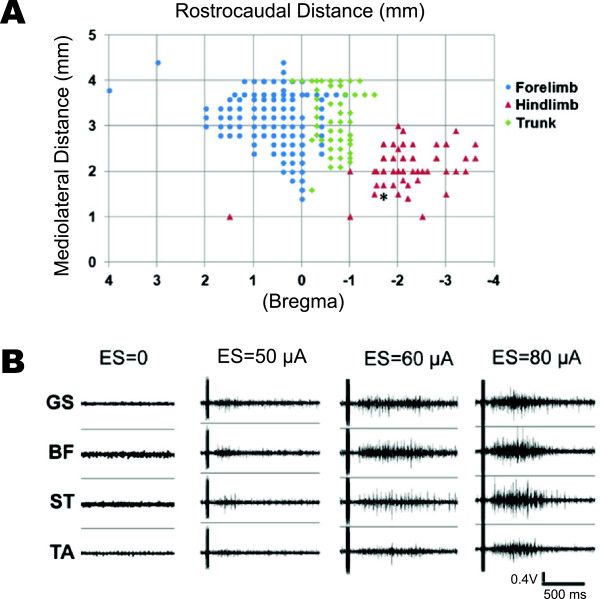
**M1 mapping in anesthetized guinea pigs. A**. Forelimb, trunk, and hindlimb representations. Star: stimulation site of panel **B**. **B**. EMG signals of four hindlimb muscles stimulated by varied currents (0–80 μA). ES: electrical stimulation, GS: gluteus superficialis, BF: biceps femoris, ST: semitendinosus, TA: tibialis anterior.

We first mapped the M1 of guinea pigs under anesthesia, focusing on the forelimb, trunk, and hindlimb areas (Figure 3A). The hindlimb area ranged from 1.5–3 mm lateral from the midline and 0.5–3.5 mm posterior to bregma, whereas the forelimb area ranged from 1.5–4 mm lateral from the midline and 0–2 mm anterior to bregma. ICMS current threshold was ~60 μA. EMG activity was recorded in four hindlimb muscles above threshold stimulating currents of 60 μA (Figure 3B). As ICMS increased from 60 to 80 μA, the amplitude and duration of EMG signals increased (Figure 3B). Evoked movements of the forelimb, trunk, or hindlimb were also taken into consideration when identifying cortical regions of interest.

### M1*“intent”* signals during treadmill walking

**Figure 4:**
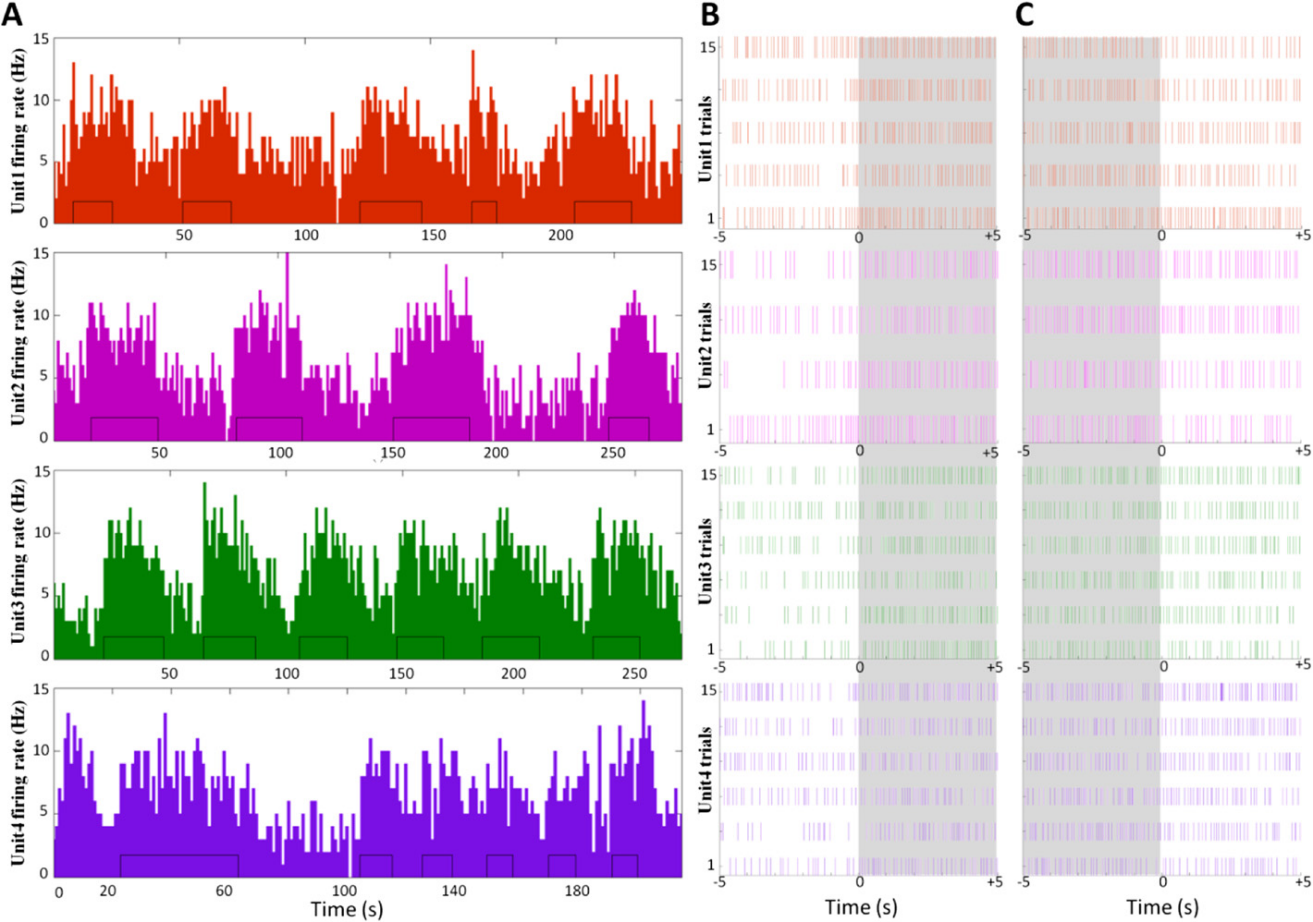
**M1 *“intent” *signal in guinea pigs during treadmill walking. A**. Single-unit firing rates during forelimb steps on a moving treadmill. The small black rectangles inside the histogram of each panel indicate individual steps. **B–C**. Raster plots for each unit showing stand-to-walk **(B)** and walk-to-stand **(C)** transitions. The shaded areas indicate time periods immediately following the commencement of steps.

Next, we recorded neural spike activity from different hindlimb area M1 neurons in eight guinea pigs during treadmill walking (Figure 4). We identified four single units in different animals with activity corresponding to forelimb locomotion during the treadmill ON and OFF phases at different treadmill speeds (Figure 4A). These neurons showed firing rates between 0 and 15 Hz. Of these neurons, the activity of unit #3 best matched the forelimb steps taken by the guinea pig, although all units showed clear patterns of activity matching patterns of forelimb locomotion. For stand-to-walk transitions, spike activity of each unit increased during the 5 s after commencing steps (Figure 4B, shaded area; unit #2 showed an obvious change). Likewise, for walk-to-stand transitions, spike activity of each unit decreased during the 5 s after completing steps (Figure 4C, unshaded area; Unit #1 and 2 showed obvious changes).

### Spinal stimulation directly driven by cortical recordings

**Figure 5:**
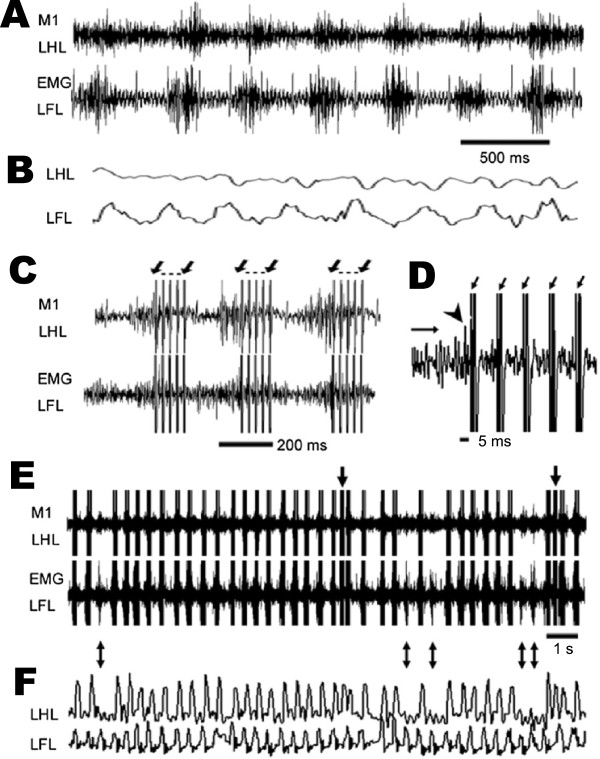
**Stimulation of LMNs from cortical signals in guinea pigs during treadmill walking at 11.1 cm/s. A**. One-channel extracellular left hindlimb (LHL) M1 signal (upper) and left forelimb (LFL) EMG signal (lower) during treadmill walking while the stimulator was switched off. **B**. Movements of LHL and LFL. **C**. Recordings of M1 (upper) and LFL EMG (lower) signals while the stimulator was switched on. Electrical stimulation was apparent as artifacts in the recording electrodes, indicated by arrows. **D**. Zoomed view of M1 recording channel. The trigger threshold was set at 0.2 V, indicated by a horizontal arrow. The neural signal triggering electrical stimulation is indicated by an arrowhead. **E**. Representative 15-s period of M1 and EMG recordings. **F**. Corresponding LHL and LFL movements.

To record cortical signals corresponding to voluntary movement, we trained three guinea pigs to walk on a treadmill with their forelimbs. Neural signals in the M1 were recorded in real-time by our *“Motolink”* hardware. The neural activity of one channel was selected and used as a triggering signal. When guinea pigs walked at a speed of 11.1 cm/s, we observed rhythmic neural activity in the left hindlimb region of the M1 that followed left forelimb EMG signals by ~100 ms (Figure 5A). As the spinal cord was not yet stimulated, hindlimb movements were passive and indistinct (Figure 5B). After the stimulator was turned on, neural signals were detected and decoded, triggering electrical stimulation (5-pulse train, 91 Hz) of the spinal cord (Figure 5C). Although stimulation artifacts were present in the recording channel, the *“Motolink”* processor ignored these artifacts by applying a delay in its spike-counting algorithm. The triggering threshold was set to achieve a reasonable correlation between left forelimb and hindlimb movements (0.2 V in Figure 5D, indicated by arrowhead). An interval of 5 ms was set between detection of the neural *“intent”* signal and stimulation of spinal cord neurons.

We observed hindlimb movements in response to spinal cord stimulation, with left forelimb movements preceding those of left hindlimbs by 92 ± 23 ms (*n* = 3). During a representative 15-s period of recording/stimulating and treadmill walking at 11.1 cm/s (Figure 5E-F; Additional file 1), the left forelimb took 39 steps, but the M1 electrode failed to detect the “intent” signal in 5 instances, resulting in a corresponding absence of 5 left hindlimb movements (double-headed arrows). These steps occurring but not detected were false negatives, which were due to insufficient amplitude of M1 activity of some forelimb stepping. Also, two odd electrical stimulations were observed during this recording/stimulating period (Figure 5E-F, single-headed arrows), for which the left forelimb took one step but the M1 electrode detected two “intent” signals, resulting in additional movement of the left hindlimb, which was considered as false positives. It came from the detection of spontaneous activities from M1. The neural activity from another guinea pig was consistently observed when it walked at a speed of 11.1 cm/s (Additional file 2: Figure S1).

**Figure 6:**
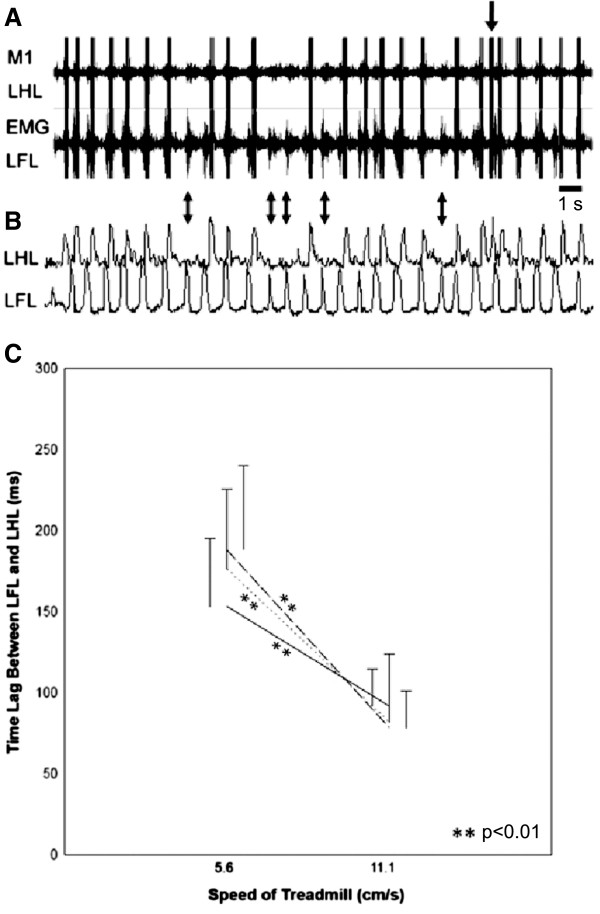
**Stimulation of LMNs from cortical signals in guinea pigs during treadmill talking at 5.6 cm/s. A**. Representative 17-s period of M1 and EMG recordings. **B**. Corresponding LHL and LFL movements. **C**. Time lag of intact LFL and stimulated LHL movements at different treadmill speeds. Data points were sampled from three animals (*n* = 10 per animal) and compared between different speeds within animals (**p < 0.01, *t*-test).

When treadmill speed was slowed to 5.6 cm/s, stepping rhythm became almost half of that at 11.1 cm/s, and M1 neural activity followed left forelimb EMG signals by ~200 ms (Figure 6A-B). After the stimulator was turned on, cortical signals reaching a preset threshold voltage triggered electrical stimulation of the spinal cord. Movement of the left forelimb preceded movement of the left hindlimb by 153 ± 42 ms (*n* = 3). During a representative 17-s period, 5 of 28 left forelimb steps were not detected by the M1 electrode (Figure 6A-B, double-headed arrows; Additional file 3). One odd electrical stimulation was observed (Figure 6A, single-headed arrow).

Regular neuronal firings were consistently observed in the hindlimb regions in the motor cortex, after the spinal transection. The M1 activities in HL preserved their function to control the HL, although their actual descending connection had been lost. The time lag between intact forelimb and stimulated hindlimb movements significantly shortened when treadmill speed increased from 5.6 to 11.1 cm/s (Figure 6C; *n* = 3 *p* < 0.01), suggesting greater neural activity rhythm in the M1 at faster walking speeds. This reduced time lag between forelimb and hindlimb movements indicates that the triggered signal did not originate from the forelimb region of the M1, sensory feedback, or other body part movements.

### Histological analysis

**Figure 7:**
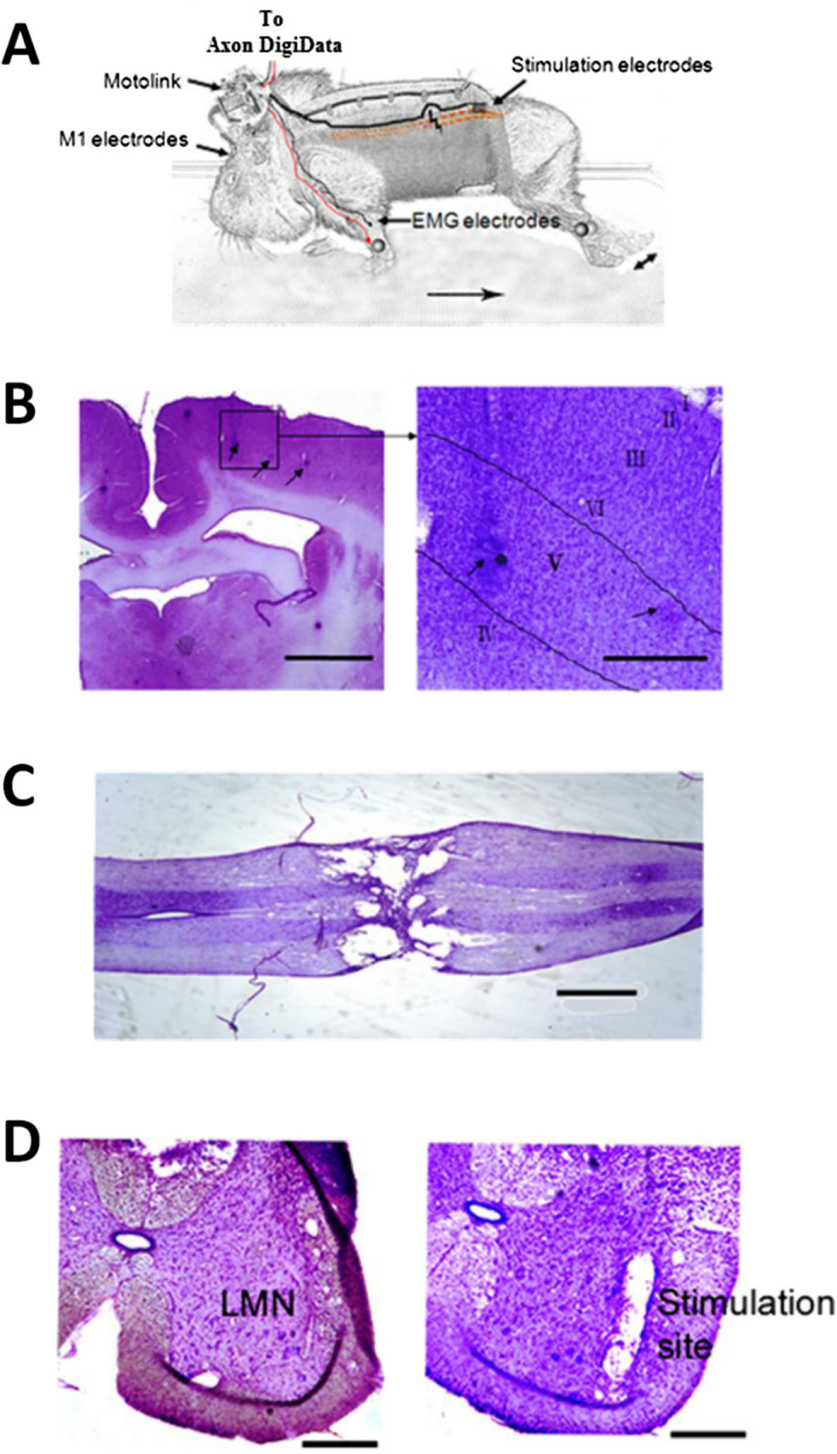
**Recording and stimulating sites. A**. Schematic drawing of experimental set-up. Triggered movement of the left hindlimb is indicated by a double-headed arrow. **B**. Recording sites in layer V of the M1. Left: Recording electrode traces (arrows). Scale bar = 1 mm. Right: Higher magnification of electrode traces. Scale bar = 200 μm. **C**. Spinal cord lesion 21 days after transection. Scale bar = 1 mm. **D**. Left: LMNs in the ventral horn of the spinal cord at L3 level. Right: Stimulating electrode trace in the spinal cord. Scale bar = 200 μm.

At the end of the experiment, we checked the placement of electrodes in the M1, the location of spinal cord transection, and the location of stimulation sites in the spinal cord (for experimental set-up, see Figure 7A) using Nissl staining. Recording electrode tips were located in layer V of the M1 (Figure 7B, right panel). Posterior to the spinal cord lesion site (Figure 7C), the stimulating electrodes were implanted in the ventral horn of the spinal cord (Figure 7D) to stimulate LMNs. These staining results confirm that neural signals were recorded from the M1 and that spinal cord LMNs were stimulated.

## Discussion

The goal of this proof-of-concept study was to test the ability of *“Motolink”* to bypass spinal cord injury and create a direct functional connection between upper motor neurons in the M1 and LMNs in the spinal cord in guinea pigs. Our low-noise, high-gain amplifier recorded these neocortical signals, which were sent to a programmed microprocessor that generated an electrical pulse train directly stimulating lumbar spinal cord motor neurons, thereby activating hindlimb muscles. Similar work has been done to restore upperlimb function [54]. As this was a proof of concept study, only one electrode in the multi-electrode array was selected to detect neuronal signals from the hindlimb region of the right M1, and stimulation was applied through one electrode in the left ventral horn, which is the location of LMNs that innervate the left hindlimb. Instead of using constant current as stimulation, we used a constant voltage of 3–7 V. As the impedance of the stimulating electrode was 50–100 kΩ, the estimated stimulation current was 30–120 μA, which is comparable to the intraspinal stimulation currents used in other studies [21,22] and the cortical stimulation currents used in our previous studies [55,56]. As our stimulation current was relatively weak, possible electrical stimulation from the reference electrode (placed at the L5 epidural surface) was unlikely to effectively stimulate spinal cord motor neurons, as has been shown in previous studies [39,57]. We used trains of five monopolar pulses (91 Hz) to allow us to distinguish stimulated hindlimb twitching. We also tested other configurations such as ‘5 pulses, 40Hz’, ‘5 pulses, 167Hz’, ‘5 and 10 pulses, 91Hz’ in pilot studies, whereas ‘5 pulses, 91Hz’ has the best effect on generating hind limb locomotion.

The insertion of electrodes into the cortex causes both acute and chronic damage to brain tissue, which is an unsolved issue in chronic experiments [58]. However, our recording electrodes, which are made of Teflon-insulated tungsten, are biocompatible and thus less harmful to brain tissue. In chronic experiments, the gradual changing of electrode impedance across days may necessitate an increase in stimulation voltage. Also, the ability to record spike activity is often lost a short period after electrode implantation [59]. At present, studies on *“Motolink”* have been limited to one month because almost no meaningful signals can be acquired from electrodes after this length of time. However, recent encouraging evidence for reduced reactions to and greater long-term functional stability of implanted electrode arrays raise hope for using neural prosthetic devices for months to years [60].

In this study, we recorded EMG signals from two groups of muscles. The gluteus superficialis, biceps femoris, semitendinosus, and tibialis anterior were four superficial hindlimb muscles used in acute experiment for mapping hindlimb region of M1. We found that supra-threshold stimulation consistently resulted in EMG activity of these four muscles. In some cases, ISMS required high voltages (e.g., 7 V) for generating hindlimb movement, possibly due to high impedance of some stimulating electrodes. EMG recordings of these muscles were primarily used to map the hindlimb region of the M1, as EMG activity allowed better resolution than visual observations of hindlimb movements. In chronic experiments, forelimb muscle triceps brachii was used to insert EMG electrodes but not hindlimb muscle. This was because forelimb stepping could be observed from treadmill walking and hindlimb activities of M1 followed and could be predicted by forelimb activities.

A future goal of this line of research is to use multichannel *“Motolink”* to record signals from ensembles of cortical neurons in real-time, which can be transferred to a group of microstimulators that activate motor neuron pools in the spinal cord below the lesion site, thereby reanimating paralyzed hindlimbs to produce smooth and graceful movements. Non-linear conversion learning between the input and output functions of LMNs is currently under consideration, and neural network control algorithms will be explored for better decoding of neural activity. Also, an effective microstimulation program may be developed to utilize cortical commands to produce natural and smooth hindlimb stepping in animals with SCI. A wireless version of the stimulator would facilitate this research [61]. Furthermore, sensory feedback control through intact reflex arcs could be utilized to regain movements, and visual feedback could be used along with proprioceptive inputs to relearn motor skills.

Our method of bypassing the site of injury to reconnect the brain and spinal cord has advantages over using EMG signals to trigger stimulation of hindlimb movement, as multichannel electrodes in the M1 should be able to acquire more detailed movement signals in a much more sophisticated manner. If these cortical signals can be connected to corresponding LMNs, animals may be able to perform coordinated movements. However, such a system would require a non-linear processor linking the multichannel recorded cortical signals to the multichannel stimulator.

Quadrupedal walking depend on posture and intralimb coordination [62,63], and thus may have influences in cortical signals between different limb areas, we tried to eliminate the possibility of one’s influence into another by carefully selecting the electrodes by intracortical microstimulation. We assumed that the cortical recording would be solely from hindlimb area and thus for hindlimb movements, not forelimb. We found that the time lag between the forelimb and hindlimb movements shortened when treadmill speed was increased, showing that we accurately implanted the recording electrode into the hindlimb region of the M1. If the *“intent”* signal from the recording electrode was actually due to crosstalk from sensory forelimb feedback or the movement of other body parts, this time lag would not have decreased in proportion to an increase in treadmill speed. Thus, the “intent” signal most likely did not originate from the forelimb region of the M1. A further investigation on the kinematic data of FL and HL in normal animal during treadmill locomotion at 5.6 and 11.1 cm/s speeds would add further evidence to the above claim.

Instead of stimulating spinal cord motor neurons to induce hindlimb movements, we could have directly stimulated the individual muscles. An advantage of directly stimulating the muscles would be better selectivity, as different muscle groups are naturally separated. As motor neurons controlling different muscle groups are very close to each other in the spinal cord, it is challenging to selectively stimulate different motor neurons with our current system. An electrode array with a three-dimensional design and low stimulation current could possibly provide a solution to this problem. However, an advantage of stimulating the spinal cord is that muscle fatigue may be decreased [64-66], as muscles are activated via physiological innervation and not artificial electrical stimulation.

Epidural spinal cord stimulation (ESCS) works by alleviating the overall excitability of spinal networks [67], whereas intraspinal microstimulation (ISMS) produces direct stimulation generated evoked movements [68]. ESCS mainly relies on combined effects of low intensity excitability of spinal neurons and their network along with different afferent inputs [69]. In contrast, ISMS activates selected motor neuron in the spinal cord to generate motor responses [20]. Thus, carefully selecting sequence of ISMS should produce an animated limbic movement. Both ESCS and ISMS hold great potentials of restoring motor functions in the paralyzed; however both miss critically the intention information to activate the stimulator. At the moment, both ESCS and ISMS are externally controlled by an operator. In real prosthetic application one should be able to control these stimulations from his/her natural “intent”. Hence, an artificial spinal bridge could provide the “intent” information and thus trigger the stimulation accordingly. With ISMS, the lower motor neurons could be directly activated following the cortical input. It would also leave the learning capability intact in the motor cortex for future motor skill acquirement.

## Conclusion

In conclusion, we developed an advanced technique of using cortical activity related to forelimb stepping to directly stimulate LMNs in the spinal cord, thereby producing stepping-like hindlimb movement in guinea pigs with spinal cord transection. This direct *“intent”*-driven system is an important first step in building a complete electronic spinal bridge to restore movement after SCI. This one-channel, one-way connection between cortical signals and LMN stimulation was shown to be effective, albeit limited in fine movement control and long-term maintenance.

## Abbreviations

SCI: Spinal cord injury; M1: Primary motor cortex; LMN: Lower motor neuron; FES: Functional electrical stimulation; ICMS: Intracortical microstimulation; ISMS: Intraspinal microstimulation; EMG: Electromyography.

## Competing interests

The authors declare no competing interests.

## Authors’ contributions

YL and JH designed the study; JH and MA developed the hardware; YL, GS, KT, and MA conducted the experiments; YL, MA, and JH analyzed the data and prepared the results; YL, MA, and JH wrote the manuscript. All authors read and approved the final manuscript.

## Supplementary Material

Additional file 1 Video demonstrating a guinea pig with spinal cord transection walking on a treadmill at 11.1 cm/s. Click here for file

Additional file 2: Figure S1 Stimulation of LMNs from cortical signals in another guinea pig during treadmill walking at 11.1 cm/s. Stimulation of LMNs from cortical signals in another guinea pig during treadmill walking at 11.1 cm/s. A. One-channel extracellular left hindlimb (LHL) M1 signal (upper) and left forelimb (LFL) EMG signal (lower) during treadmill walking while the stimulator was switched off. B. Recordings of M1 (upper) and LFL EMG (lower) signals while the stimulator was switched on. Electrical stimulation was apparent as artifacts in the recording electrodes, indicated by arrows. C. Zoomed view of M1 recording channel. The trigger threshold was set at 0.2 V, indicated by a horizontal arrow. The neural signal triggering electrical stimulation is indicated by an arrowhead. Click here for file

Additional file 3 Video demonstrating a guinea pig with spinal cord transection walking on a treadmill at 5.6 cm/s. Click here for file
