# Supplementary figures and images for: Electronic bypass of spinal lesions: activation of lower motor neurons directly driven by cortical neural signals
Source: J Neuroeng Rehabil. 2014 Jul 3;11:107. doi: 10.1186/1743-0003-11-107 (PMC4094416; doi:10.1186/1743-0003-11-107)

**a**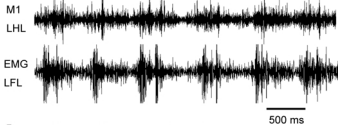**b**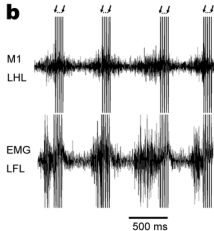**c**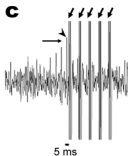

Supplement: Additional file 2: Figure S1 — Stimulation of LMNs from cortical signals in another guinea pig during treadmill walking at 11.1 cm/s. Stimulation of LMNs from cortical signals in another guinea pig during treadmill walking at 11.1 cm/s. A. One-channel extracellular left hindlimb (LHL) M1 signal (upper) and left forelimb (LFL) EMG signal (lower) during treadmill walking while the stimulator was switched off. B. Recordings of M1 (upper) and LFL EMG (lower) signals while the stimulator was switched on. Electrical stimulation was apparent as artifacts in the recording electrodes, indicated by arrows. C. Zoomed view of M1 recording channel. The trigger threshold was set at 0.2 V, indicated by a horizontal arrow. The neural signal triggering electrical stimulation is indicated by an arrowhead. [file 1743-0003-11-107-S2.pdf]
